# Investigating disater risk management and climate change adaptation effectiveness in freetown, Sierra Leone

**DOI:** 10.4102/jamba.v17i1.1904

**Published:** 2025-08-28

**Authors:** Edwin Sam-Mbomah, Ksenia Chmutina, Alister Smith, Susie Goodall, Lee Bosher

**Affiliations:** 1 Department of Water Engineering Development Centre, School of Architecture, Building and Civil Engineering, Loughborough University, Leicestershire, United Kingdom; 2 Institute of Environmental Management and Quality Control, School of Environmental Sciences, Njala University, Freetown, Sierra Leone; 3 Department of Civil Engineering, School of Architecture, Building and Civil Engineering, Loughborough University, Leicestershire, United Kingdom; 4 Department of Research Excellence, School of Business, University of Leicester, Leicester, United Kingdom

**Keywords:** disaster risk management, climate change adaptation, hazard, vulnerability, Freetown, Sierra Leone

## Abstract

Freetown, Sierra Leone, faces increasing risks from hazards such as floods, landslides, mudslides, fires and coastal erosion, threats that are increasingly intensified by climate change. Although disaster risk management (DRM) and climate change adaptation (CCA) policies are in place, concerns persist regarding their effectiveness. This study investigates the extent to which these policies have transitioned from good intentions to effective interventions in Freetown. Employing a case study approach, the research critically examines the disaster context, reviews relevant policy frameworks and implementation processes, and identifies both enablers and barriers to effective policy execution across five informal settlements, Kroo Bay, Susans Bay, Kulvert, Kolleh Town and Dwarzack. The findings reveal systemic deficiencies in DRM and CCA, including weak legal and institutional structures, poor inter-agency coordination, inadequate enforcement, limited access to data, and ineffective risk communication. Further obstacles include insufficient community engagement, the absence of legal mechanisms for resolving DRM and CCA related disputes, corruption, political instability, underinvestment in research and technology, and inadequate urban planning. The study recommends strengthening policy and institutional frameworks, promoting integrated collaborative planning processes, and supporting community-led resilience initiatives. Key priorities include enhancing risk assessments, developing robust early warning systems (EWS), improving urban planning, and fostering innovation through research and technology.

**Contribution:**

The study identifies key measures for effective DRM and CCA in Freetown, including community inclusion, capacity building, financial mechanisms, data management, risk communication, and resilient infrastructure development, especially for “at-risk” communities.

## Introduction

Sierra Leone, a coastal West African country, is acutely vulnerable to the impacts of climate change. Its capital city, Freetown, regularly experiences climate-induced hazards such as floods, heatwaves and landslides, which significantly affect its socio-economic and environmental systems. As one of the world’s poorest countries, Sierra Leone faces major challenges in disaster preparedness and response, largely because of limited domestic resources and a high reliance on donors (UNDP 2019). The recurrent occurrence of floods and landslides in Freetown continues to disrupt daily life, displacing families, destroying property and undermining livelihoods (Cui et al. 2019).

This persistent vulnerability underscores the urgent need for effective disaster risk management (DRM) and climate change adaptation (CCA) strategies. Freetown’s exposure to climate-related hazards demands a focused understanding of the local drivers, impacts and institutional responses. Such insights are critical for informing national policies and international interventions aimed at strengthening resilience. Moreover, aligning these strategies with global frameworks, such as the Sendai Framework for Disaster Risk Reduction (SFDRR) and the Paris Agreement, is essential for ensuring coherent and sustainable adaptation efforts (Sawaneh, Fan & Sesay 2024). Freetown is increasingly vulnerable to climate-induced hazards such as flooding and landslides. These risks are exacerbated by rapid urbanisation, widespread deforestation and inadequate infrastructure, particularly within informal settlements. In this context, integrating DRM and CCA is essential for enhancing urban resilience. Such integration addresses overlapping vulnerabilities and promotes sustainable urban development and risk-informed planning (World Bank 2021).

Mainstreaming CCA into DRM strategies offers a pathway to proactively manage climate variability while mitigating the impacts of natural hazards. Achieving this requires coordinated efforts across multiple stakeholders, the establishment of strong policy and institutional frameworks and sustained capacity-building efforts. Prioritising integrated approaches can significantly reduce socio-economic losses, protect livelihoods and foster inclusive, climate-resilient development. Lessons from Freetown are increasingly relevant for other urban centres facing similar climate and development pressures (World Bank 2017).

In Sierra Leone, government agencies such as the National Disaster Management Agency (NDMA) and the Environment Protection Agency of Sierra Leone (EPA-SL), working through the National Climate Change Secretariat (NCCS), are actively engaged in strengthening disaster preparedness and CCA initiatives. Against this backdrop, this study investigates the effectiveness of DRM and CCA policies in Freetown, with a particular focus on their progression from policy intentions to tangible, on-the-ground outcomes.

The study examines Freetown’s disaster risk profile and reviews the institutional and policy frameworks guiding DRM and CCA efforts. It explores both barriers and enablers that influence the implementation of these policies, especially in marginalised communities characterised by high poverty and limited infrastructure. It aims to identify practical, context-specific strategies to close the gap between policy aspirations and effective, community-level interventions.

## Overview of disaster risks in Sierra Leone

### Overview of Freetown

Freetown, the capital of Sierra Leone, lies on a mountainous peninsula and is home to over 1 million people. The city’s steep topography and ancient geological formations dating back approximately 202 million years make it particularly vulnerable to environmental hazards. Rapid urbanisation, coupled with unplanned development, has led to the expansion of informal settlements and widespread deforestation. These factors significantly increase the city’s exposure to risks such as flooding and landslides. Climate change further intensifies these threats by driving extreme weather events that jeopardise infrastructure, livelihoods and economic stability (Koroma et al. 2021; Redshaw et al. 2019; Umeji 1983; Wadsworth & Lebbie 2019).

Freetown experiences a tropical climate, with a dry season from November to April and a wet season from May to October. Heavy rainfall during the wet season heightens the likelihood of flash floods and landslides. Recent shifts in monsoon patterns combined with anthropogenic pressures such as deforestation and inadequate solid waste management have disrupted the city’s natural hydrology and drainage systems. These disruptions contribute to rising urban temperatures and increased environmental degradation, amplifying the impacts of climate change on water flow and extreme weather events (Dodman et al. 2022; GoSL 2022a).

While Freetown boasts of rich social and cultural diversity, it also contends with systemic challenges common in least developed countries (LDCs), including high poverty levels, inadequate housing and limited access to essential services. Although various organisations are engaged in social development, youth empowerment and economic diversification, structural issues such as extreme poverty, income inequality and weak governance remain pervasive. These challenges are compounded by the effects of rapid urban growth and widespread youth unemployment (Adrian et al. 2019; Allen et al. 2017; Cui et al. 2019; GoSL 2018).

Freetown has experienced numerous disasters resulting from both natural and human-induced hazards. Hydrometeorological threats such as flooding, storms, lightning, droughts, mud and landslides occur frequently and often cause widespread damage. Human-induced hazards, including fire outbreaks, are typically localised but occur with high frequency. Technological hazards, such as transport-related explosions and accidents, tend to be occasional and site specific. Civil unrest, while infrequent, can have widespread impacts when it does occur. Additionally, the city is vulnerable to biological hazards, including epidemics and pandemics, which have the potential for nationwide impact (EM-DAT 2023).

### Case study location profile

**FIGURE 1:**
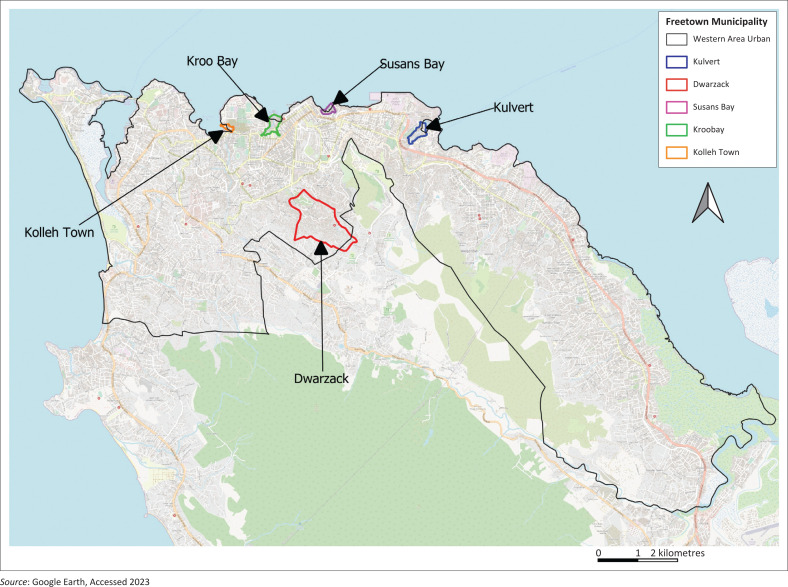
Map of Freetown indicating study areas.

The study focuses on five selected communities within Freetown, namely Kroo Bay, Susan’s Bay, Kulvert, Kolleh Town and Dwarzack (Figure 1). These areas were chosen because of their distinct characteristics in terms of hazard exposure, levels of vulnerability and local community strategies for disaster mitigation and response. The methodological details underpinning this selection are discussed in the methodology section.

**FIGURE 2:**
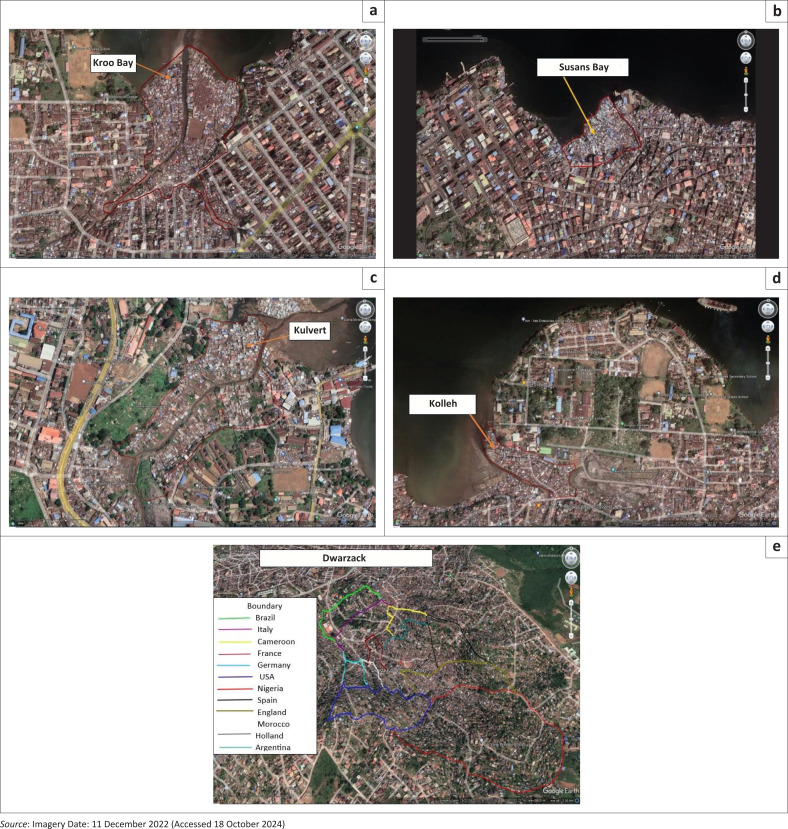
Google Earth Map of Kroo Bay (a), Susan’s Bay (b), Kulvert (c), Kolleh Town (d) and Dwarzack (e) communities.

Kroo Bay, named after the Kroo ethnic group, is a densely populated informal settlement in central Freetown (8°29’N, 13°14’W), situated 39 metres above sea level, with an estimated population of 18 000 (SLURC, 2018). Susan’s Bay, located along the Atlantic Ocean (8°29’30”N, 13°13’47”W), lies at an elevation of 77 metres and is home to approximately 7041 residents (UNOPS 2023). Kulvert, formerly known as Lower Racecourse, lies east of the city (8°29’09.25”N, 13°12’21.97”W), with a population of 9193 (Caritas 2022). Kolleh Town (8°29’10.10”N, 13°15’06.61”W), adjacent to the Kingtom dumpsite, has around 5200 residents (SLURC 2018). Dwarzack (8°28’33.94”N, 13°13’59.65”W), established in 1914, is a hillside community located in central Freetown, with an estimated population of 16 500 (SLURC 2018). Figure 2 provides a visual overview of these communities using Google Earth imagery (Caritas 2022; Mapcarta 2024; SDI 2015; SLURC 2018).

Kroo Bay, Susan’s Bay, Kulvert and Kolleh Town are low-lying coastal informal settlements, while Dwarzack is situated on hilly terrain. All five communities face serious development challenges, including inadequate infrastructure, poor waste management, insufficient sanitation services and high exposure to hazards such as flooding, landslides, rockfalls and fires. Practices such as ‘banking’ (land reclamation using solid waste) and informal land occupation (land grabbing) exacerbate environmental degradation, heightening the risk of disasters (CODOHSAPA & Federation of Urban and Rural Poor [FEDURP] 2011; SLURC, 2017; 2018).

Despite these vulnerabilities, the communities display remarkable resilience through strong social cohesion, active community-based organisations (CBOs) and informal governance mechanisms that support DRM and CCA. Livelihoods are diverse, ranging from petty trading and artisanal skills to informal maritime activities conducted under the cover of night, often because of limited access to formal employment (Caritas 2022; CODOHSAPA & FEDURP 2011; SLURC, 2017).

### Institutional structural framework for disaster risk management and climate change adaptation in Sierra Leone

Sierra Leone’s DRM and CCA frameworks are grounded in multi-stakeholder collaboration involving government agencies, local authorities, international partners, non-governmental organisations (NGOs), CBOs and local communities. These actors work collectively to reduce disaster risks and strengthen community resilience, aiming to protect lives, livelihoods and infrastructure. Notably, institutional arrangements for both DRM and CCA were developed around the same period, supported by evolving policy frameworks that facilitate coordination, resource allocation, standard-setting, integration, community participation, monitoring and accountability (Bang 2022). Initially established in 2002, the Office of National Security (ONS) was tasked with disaster management functions. However, its broad focus on national security limited its effectiveness in managing disasters, specifically prompting the formulation of the National Disaster Management Policy in 2006 (Turay & Gbetuwa 2022). To address these limitations, the NDMA was created in 2020 as the lead agency for DRM, operating under the Office of the Vice President. The National Disaster Risk Management (NDRM) coordinates multi-sectorial efforts across government departments, NGOs and international stakeholders.

The national coordination structure includes the NDRM platform (Platinum Level), chaired by the Vice President and serving as the highest advisory body on disaster issues. This platform reports to the National Security Council (NSC), which is led by the President. Beneath the NDRM is the National Strategic Situation Group (NSSG) (Gold Level), also led by the NDMA and chaired by the Director-General, and is responsible for managing disaster response operations. At the operational level, relevant ministries, departments and agencies (MDAs) engage through the Silver Level, providing tactical and logistical support (GoSL 2020). Although the 2006 policy is in draft, the NDMA still relies on it. The draft policy outlines five key priorities and nine strategic areas, including political leadership, stakeholder engagement and public awareness. A revised DRM policy, developed in 2021, is currently awaiting parliamentary approval (GoSL 2020).

On the climate side, the EPA-SL, established in 2008, is responsible for environmental regulation and climate action. Its work is guided by the National Climate Change Policy (NCCP) and *the 2022 Climate Change Act*. The EPA-SL hosts the NCCS, which leads national climate initiatives, including policy development, stakeholder consultations, enforcement and representation in global negotiations. These functions are supervised by the Ministry of Environment and Climate Change (MoECC) (GoSL 2022b).

The 2021 NCCP provides strategic direction for climate action, encompassing adaptation, mitigation and resilience building for sustainable development. It aligns with the Paris Agreement and promotes environmental sustainability through eight core policy priorities and 10 directives aimed at reducing climate risks and enhancing national resilience (GoSL 2021a; 2021b).

**FIGURE 3:**
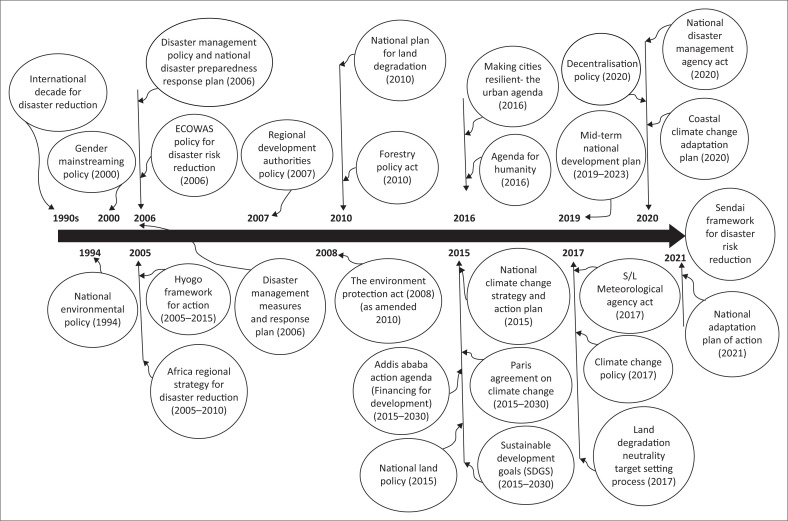
Disaster risk management and climate change adaptation policies in Sierra Leone and linkage to international policy.

As illustrated in Figure 3, Sierra Leone’s DRM and CCA policy evolution reflects its commitment to both national and international frameworks, including the Medium-Term National Development Plan (MTNDP) (2019–2023), the Sendai Framework for Disaster Reduction and the Paris Agreement. Through the leadership of the NDMA and EPA-SL, supported by stakeholders, the country is working to mainstream DRM and CCA into national development planning. Key priorities include improving public awareness, securing access to climate and disaster funding and strengthening the institutional capacity of local communities to respond effectively to climate and disaster-related challenges (GoSL 2018, 2020; UNDRR 2015).

## Methodology

The study adopted an exploratory qualitative research design grounded in a philosophical stance of relativism and employed a case study strategy to examine the effectiveness of DRM and CCA policies in informal settlements in Freetown, Sierra Leone. An inductive approach was used to uncover participants’ perspectives and patterns, generating empirical insights to inform potential policy reforms for more effective DRM and CCA implementation.

**FIGURE 4:**
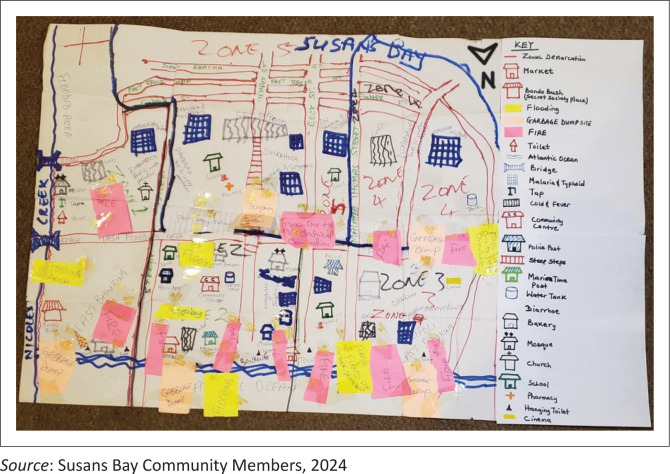
In-field community participatory hazard, vulnerability and capacity map of Susan’s Bay community.

Data collection involved multiple qualitative methods to ensure rigour and richness. These included:

- In-depth policy analysis to identify existing policy gaps.
- Community participatory hazard, vulnerability and capacity (HVC) mapping exercises, conducted with local residents and CBOs in five disaster-prone communities, namely Kroo Bay, Susan’s Bay, Kulvert, Kolleh Town and Dwarzack, used to document local hazards, vulnerabilities and capacities (see Figure 4).
- Semi-structured interviews with a wide range of stakeholders.
- Direct field observations.

A total of 22 in-person semi-structured interviews were conducted between January and April 2023. Each session lasted approximately 60 min and included participants such as:

- Policymakers
- Representatives from research institutions (e.g. SLURC)
- Community-based organisations (e.g. FEDURP and Community Disaster Management Committee [CDMC])
- International organisations (e.g. UNDP and UNOPS)
- Non-governmental organisations (e.g. Sierra Leone Red Cross Society [SLRC])
- Members of local communities

Interviews with policymakers, research institutions and representatives of NGOs and international organisations were conducted in English to ensure clarity and fluency. Interviews with CBOs and community members were conducted in the Krio language to enhance participant comfort and expression. This multilingual approach enriched the dataset with subjective insights and contextual understandings. The flexible interview format enabled participants to share detailed responses and lived experiences (Dunwoodie, Macaulay & Newman 2022).

Field observations further complemented the interviews by capturing both physical and social dynamics within communities. This helped to identify unrecorded elements such as local early warning systems (EWS), coping strategies and community-led response mechanisms.

### Selection criteria for stakeholders

Community stakeholders were nominated by their respective communities to represent different zones in the HVC mapping process. These stakeholders participated in pilot meetings facilitated by the chairpersons of two CBOs: the FEDURP and the CDMC. Communication during the process was conducted in Krio to ensure inclusivity.

Communities were prioritised based on key criteria including geographic location, population size, socio-economic diversity, existing DRM and CCA capacities and their willingness to participate actively. This inclusive approach ensured that local perspectives, priorities and lived experiences were adequately captured in the mapping process.

**FIGURE 5:**
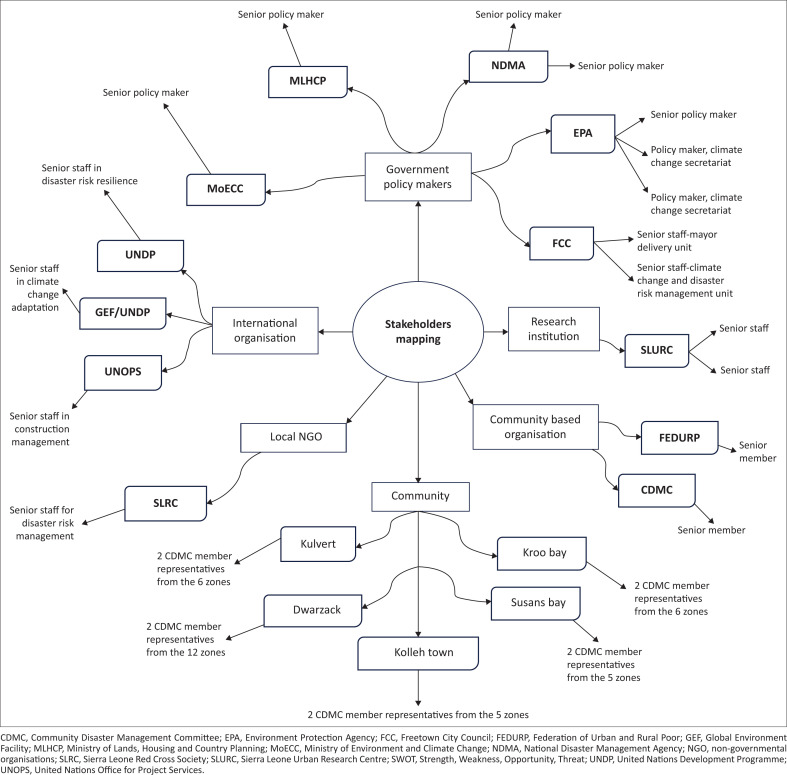
Stakeholder mapping.

Purposive sampling was employed, guided by a stakeholder mapping exercise (Figure 5), to ensure the involvement of a wide range of actors with relevant expertise in DRM and CCA. These included policymakers, researchers, NGOs, international agencies, CBOs and community leaders. Special attention was given to gender balance, age diversity, socio-economic representation and equitable power dynamics, following the ‘Power versus Interest’ framework.

### Data analysis

The data analysis process involved interpreting information from policy reviews, community HVC mapping and thematic analysis of stakeholder interviews.

A SWOT analysis was conducted to evaluate the strengths, weaknesses, opportunities and threats of DRM and CCA policies. This entailed a systematic examination of policy content, objectives, implementation strategies and potential impacts, with a specific focus on their contribution to building community resilience to hazards (Farooq 2023).

Community HVC maps were digitised using Adobe Illustrator and overlaid on Google Earth, to visualise hazard zones, exposure levels and community capacities. This geospatial analysis identified critical vulnerabilities, highlighted drainage systems and pinpointed opportunities to enhance housing infrastructure in high-risk areas.

Stakeholder interviews provided qualitative insights that complemented the policy and mapping analyses. These interviews helped identify perceived policy threats, relevance and impact (Gurel 2017). Transcriptions were used to retain participants’ perspectives while ensuring confidentiality through coded identifiers. The data were manually coded and imported into Nvivo for structured thematic analysis. Patterns and themes were identified within a predefined framework. Triangulating interviews, field observations, HVC maps, SWOT analysis and existing literature strengthened the rigour and reliability of the findings, uncovering key trends and reducing potential bias (Carter et al. 2014; Yin 2009, 2014).

## Findings

### Policy analysis

#### Disaster risk management policy analysis

Section 2.1 of Sierra Leone’s 2006 draft DRM policy mandates the NDMA to develop a national plan focused on risk assessment, EWS and proactive capacity building. Sections 4.11, 2.2.1 and 2.2.2 underscore the importance of community participation, cross-sectorial integration, stakeholder collaboration, knowledge sharing and the establishment of a legal framework that supports preparedness, mitigation, response and recovery, critical for enhancing resilience and ensuring policy compliance.

Despite a review in 2021, the DRM policy remains in draft form and unapproved though in use. This delay, along with persistent governance and institutional capacity constraints, undermines its effectiveness and inclusiveness. Key weaknesses include vague definitions of stakeholder roles, absence of clear funding strategies and poor data collection mechanisms, all of which hamper coordination, preparedness and risk assessment. The policy also inadequately addresses the specific vulnerabilities of marginalised populations, lacks provisions for translation into Indigenous languages and fails to incorporate local and traditional knowledge systems.

Although the policy recognises climate change as a critical factor, it lacks comprehensive adaptation strategies. Furthermore, it does not provide measures to safeguard policy continuity amid political instability, a recurring issue that disrupts implementation. The policy overlooks emerging disaster risks, regional disparities and the specific needs of vulnerable communities. Additionally, deficiencies in monitoring and evaluation frameworks and in disaster risk communication further weaken its relevance and effectiveness.

Nonetheless, the draft DRM policy offers important opportunities for strengthening disaster resilience. Sections 2.2.4, 2.2.6, 2.2.9 and 2.2.14 point to potential improvements in institutional coordination, technical capacity and resource mobilisation. If aligned with global frameworks, such as the Sendai Framework for Disaster Reduction, Sierra Leone could benefit from international financing mechanisms like the Green Climate Fund (GCF) and Global Environment Facility (GEF).

Regional collaboration through ECOWAS can enhance cross-border disaster preparedness, EWS and shared resources. Technology advancements such as satellite imaging and geographic information systems (GIS) can improve disaster response capabilities when effectively integrated with international partnerships. Collaboration with agencies such as the UNDP and the World Bank could provide technical assistance, capacity development and access to the best global practices. Engaging the diaspora also presents opportunities for financial support, technical skills transfer and international advocacy.

Section 2.7 further provides for global disaster advocacy campaigns to raise awareness of Sierra Leone’s vulnerabilities, which could attract additional funding and support. Incorporating DRM considerations into regional trade agreements may promote resilient infrastructure and foreign investment. Moreover, public–private partnerships (PPPs) with global businesses can drive innovation and enhance infrastructure resilience.

Despite these opportunities, several threats to DRM policy implementation persist. Participants in the study highlighted inadequate domestic funding as a critical constraint, limiting the NDMA’s ability to conduct risk assessments and engage communities. Broader external threats include global economic volatility, regional instability, shifting international policy priorities and technological capacity gaps. Additional risks involve cybersecurity threats related to the integration of new technologies, increased variability in global climate patterns, weak regional DRM systems and a heavy dependence on international technical support.

Geopolitical instability, misalignment between DRM policy and local developmental priorities because of the drive for global policy harmonisation and overreliance on international organisations for data also continue to impede the successful implementation of DRM initiatives in Sierra Leone.

#### Climate change policy analysis

The NCCP of Sierra Leone presents several strengths. It lays down a robust legal foundation for climate action by clearly defining the roles of the NCCS and strengthening its capacity to regulate and enforce climate policies. The policy promotes inclusive stakeholder participation, sustainable financing mechanisms and the mainstreaming of climate adaptation into national development strategies and the UN Sustainable Development Goals (SDGs). Additionally, it advances climate education and institutional accountability through enforcement mechanisms and commits to halving national vulnerability by 2030 through a gender-responsive and conservation-focused approach.

Despite these strengths, the NCCP faces significant implementation challenges. Limited financial resources, inadequate institutional capacity and weak inter-agency coordination hinder effective execution of adaptation measures. Enforcement remains weak, with widespread non-compliance at the community level. The policy lacks clear provisions on climate justice and equity, disproportionately affecting vulnerable and marginalised populations. Sectorial integration remains inadequate, resulting in policy silos and conflict particularly in relation to DRM. Furthermore, there is insufficient focus on gender and social equity, which perpetuates systemic inequalities. Low levels of community awareness, a lack of capacity-building initiatives and unclear monitoring and evaluation frameworks further undermine the effectiveness of the policy. Section 6.0 of the NCCP underscores the importance of strengthening climate governance to meet Sierra Leone’s Nationally Determined Contributions (NDCs) and to align with international climate objectives. Regional frameworks, such as the African Climate Change Strategy and synergies with the SDGs, especially Goals 13 (Climate Action) and 15 (Life on Land), enhance policy coherence and promote sustainable development. Section 1.1.7 highlights the country’s opportunity to enhance transparency and accountability by aligning with global agreements, including the Paris Agreement and United Nations Framework Convention on Climate Change (UNFCCC). Compliance with these agreements not only fosters global partnerships and access to technical assistance but also improves policy legitimacy and credibility.

Section 5.1.2.4 of the NCCP outlines mechanisms for accessing climate finance through institutions such as GCF, GEF and other adaptation funds. These financial instruments support Sierra Leone’s climate mitigation and resilience-building efforts. International contributions, both financial and technical, particularly from partners like UNDP and the World Bank, facilitate the integration of climate considerations into national development plans, with positive spillovers for renewable energy, disaster risk reduction and sustainable development.

Nevertheless, the policy analysis and insights from interview participants reveal a number of external threats to the NCCP’s effectiveness. Chief among these is the country’s heavy dependence on international funding, coupled with persistent gaps in global climate finance flows. Limited and inconsistent international financial support undermines implementation, monitoring and enforcement capacities. Additional external threats include weak global commitments to greenhouse gas (GHG) emission reductions, volatility in carbon markets and global economic and geopolitical instability. Moreover, limited regional cooperation and disparities in access to climate technologies and expertise present further obstacles. Shifts in global climate priorities and governance frameworks also risk diluting Sierra Leone’s ability to effectively implement its national climate policy.

### Key findings from the interviews

Thematic analysis of interviews on DRM and CCA policy frameworks in Freetown revealed significant barriers to effective implementation, despite ongoing government efforts. Respondents highlighted several persistent challenges, including weak integration between DRM and CCA policies, overall policy incoherence and fragmented institutional structures. These issues are compounded by inadequate funding, a high reliance on donor support, poor policy enforcement and limited support from the judicial system.

Additional obstacles include low levels of public awareness, minimal community engagement and insufficient risk assessments. Participants also noted gaps in monitoring, inconsistency in government commitment, corruption, political interference and general political instability. A shift in public and institutional attitudes was deemed necessary to drive progress.

Furthermore, poor coordination, weak collaboration, ineffective communication and limited data sharing among stakeholders were cited as major implementation bottlenecks. The underutilisation of research and technology and poor urban planning were also seen as critical constraints to the successful execution of DRM and CCA initiatives in Freetown.^1^

### Key findings from community hazard, vulnerability and capacity mapping

**FIGURE 6:**
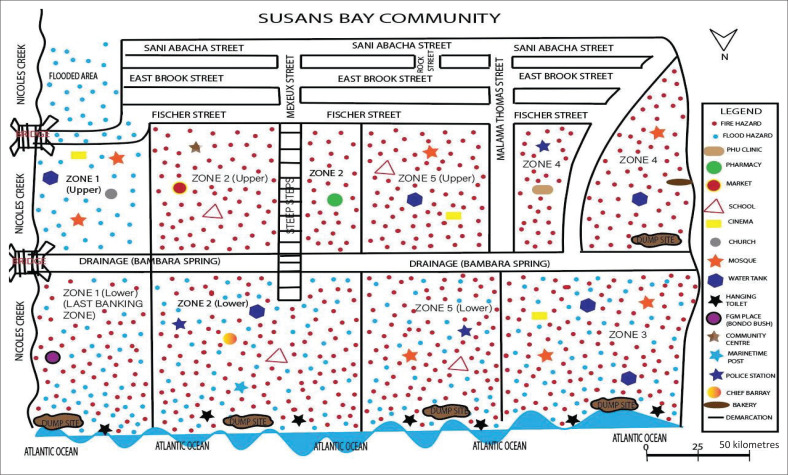
Community participatory digital hazard, vulnerability and capacity map – Susan’s Bay.

Figure 6 presents an example of the digitised HVC map for the Susan’s Bay Community. Across all mapped communities whether low-lying or located on hilltops, the findings consistently revealed significant concerns related to flooding and fire hazards.

Kulvert, a predominately low-lying area with some steep slopes, and Dwarzack, a hilltop community, are both particularly vulnerable to mudslides. In addition, Kolleh Town and Kulvert are the only selected communities exposed to smoke hazards, mainly because of their proximity to open dumpsites.

Dwarzack faces a unique threat from rolling boulders, attributed to its steep terrain and underlying granitic geology. Moreover, Kulvert is the only community exposed to chemical dust, resulting from its closeness to the cement factory in Clien Town, Freetown.

Across all five study sites, community members reported having limited capacity to mitigate these hazards effectively. This was largely because of their constrained local resources and the insufficient support received from government agencies. Notably, there was a lack of assistance in developing community-specific disaster preparedness plans and strategies to enhance local adaptation and resilience.

**FIGURE 7:**
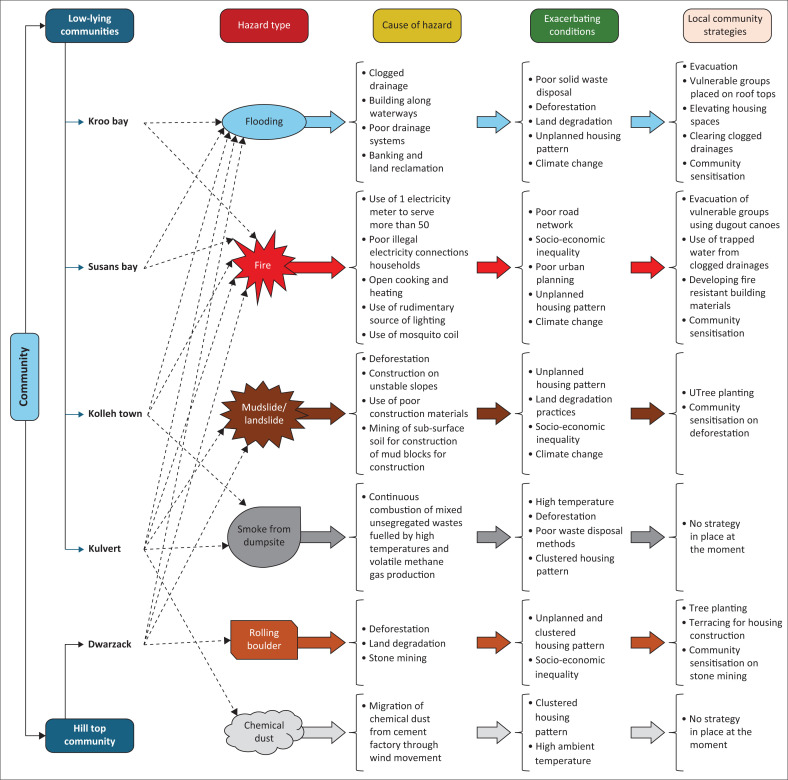
Hazard types, causes, exacerbating conditions and local community strategies in the five selected communities.

Figure 7 summarises the type of hazards identified in each community, their root causes, exacerbating conditions and the local strategies being used to cope with them:

- Community sensitisation
- Evacuation of vulnerable groups using dugout canoes
- Use of trapped water from clogged drainages
- Developing fire resistant building materials
- Community sensitisation
- Tree planting

The multi-hazard exposure of both low-lying and hill-top communities in Freetown intensified by compounding factors illustrated in Figure 7 significantly increases their vulnerability to disasters. These communities face persistent challenges because of limited capacity to prepare for, mitigate, respond to and recover hazards. This lack of capacity has entrenched cycles of loss, displacement and socio-economic hardship, particularly among marginalised populations. These findings underscore the urgent need for effective DRM and CCA policy interventions aimed at reducing vulnerability and fostering the development of sustainable, resilient communities capable of withstanding multiple hazards.

### What is required to achieve successful disaster risk management and climate change adaptation integration and implementation?

The findings reveal that Sierra Leone’s challenges in DRM and CCA do not stem from lack of policy frameworks. Instead, the core issue lies in translating existing policies into clear, actionable strategies that address the complex, interrelated and often overlooked challenges on the ground. Achieving successful implementation requires a coordinated effort to address these interconnected issues holistically.

The following discussion outlines key implications for enhancing DRM and CCA policy integration and implementation. Each is considered equally critical, and their order does not imply any hierarchy of importance.

### Implications of policy linkage between disaster risk management and climate change adaptation

The increasing frequency and severity of disasters underscores the urgent need to integrate DRM and CCA frameworks (Cardona et al. 2012). In Sierra Leone, both policy areas share the overarching goal of building resilience, yet they differ in scope. DRM focuses on hazard response and risk reduction, while the NCCP emphasises adaptation to long-term climate impacts. However, the findings indicate that there is a lack of policy coherence between the DRM and CCA policies in Sierra Leone, highlighting the need for their integration.

Aligning these policy domains can help optimise resource use, strengthen community resilience and improve overall risk governance, especially vital in resource-constrained contexts such as Sierra Leone. Effective integration requires dismantling institutional silos, improving coordination among stakeholders and enhancing mechanisms for data sharing and joint planning (Birkmann & Teichman 2015). A harmonised approach would empower institutions like NDMA and NCCS to adopt sustainable, cross-sectorial strategies that reduce vulnerabilities and protect development gains. This can be achieved by establishing a coordinated institutional framework that aligns DRM and CCA policies, planning and implementation across sectors, led by a central authority that supports collaboration among government agencies, local authorities and development partners.

### Implications of weak legislative and policy frameworks

As Shaw et al. (2017) argued, effective DRM and CCA legislation is essential for reducing disaster impacts. In Sierra Leone, particularly in Freetown, gaps in policy design and enforcement have contributed to recurrent loss of life, infrastructure damage and economic disruption. Vulnerable and marginalised communities bear the brunt of these failures because of insufficient policy support and exacerbating socio-economic inequalities.

The finding shows that as the DRM and CCA policy frameworks in Sierra Leone lack strong coherence, poor policy enforcement and non-compliance are major obstacles to effective DRM and CCA. Even though the frameworks lack coherence, enforcement can improve disaster resilience and support comprehensive policy integration. However, these challenges are driven by political interference, limited technical expertise, inadequate funding and incoherent policy structures (Thomas & Warner 2019). For example, widespread violations of building codes, especially in informal settlements in Freetown, are linked to weak governance and institutional distrust. This not only undermines community engagement but also restricts resources for preparedness, delays response mechanisms and impedes the implementation of locally relevant risk reduction strategies (World Bank 2019).

### Weak coordination and collaboration among disaster risk management and climate change adaptation stakeholders

Weak coordination between stakeholders in DRM and CCA results in fragmented engagement and inconsistent interventions, undermining the effectiveness of policies and the efficient use of resources (Abdeen et al. 2021). Although the institutional efforts by the NDMA and NCCS to support collaboration through inter-agency platforms exist, meaningful coordination remains elusive. One critical gap is the limited inclusion of community voices in decision-making processes. This exclusion hinders community ownership, leads to overlapping or duplicated initiatives and leaves some vulnerable groups without adequate support. As a result, intervention efforts become inefficient and can exacerbate existing inequalities, compounding the vulnerability of ‘at-risk’ populations (Pelling 2011). For coordination and collaboration to be effective, decision-making must be inclusive, and evidence-based information should be shared widely to align stakeholder objectives and strengthen community resilience.

### Inadequate institutional frameworks and capacity

The findings also underscore the urgent need to strengthen institutional frameworks and build capacity for DRM and CCA implementation in Sierra Leone. Institutional weaknesses within the NDMA and NCCS have resulted in siloed operations, limiting the integration of resilience into planning processes and reducing the effectiveness of resource use. These structural limitations directly affect local communities, leading to reduced participation, underutilisation of indigenous knowledge and inadequate disaster preparedness. Strengthening institutional capacity is therefore essential not only to translate policies into concrete actions but also to ensure better coordination and support community-based risk reduction.

Without improvements in institutional capacity, critical gaps in risk assessment, disaster planning and response will persist (Cardona et al. 2012; Sperling & Szekely 2021). Investments in institutional training and expertise development are necessary to ensure that personnel are equipped with skills required to manage disaster risks and enhance resilience at the community level. Howell (2018) highlighted that robust institutional capacity is foundational to effective policy implementation and meaningful community engagement. Additionally, adequate funding is crucial to support these capacity-building initiatives. With adequate investment, DRM and CCA strategies can become more proactive, better coordinated and more effectively implemented (Macarthy et al. 2024).

### Implications of weak community capacity building and engagement

Effective DRM and CCA depend heavily on strong community engagement and empowerment. Inclusive policy processes that involve communities support resilience by promoting local ownership, tapping into indigenous knowledge and encouraging cross-sectorial collaboration (Van Niekerk et al. 2018).

Findings from this study indicate that strained relationships between state institutions and communities hinder policy uptake and weaken resilience and adaptive capacities. When communities are excluded, particularly marginalised groups, decision-making becomes disconnected from local realities. This reduces the integration of valuable local knowledge into DRM and CCA strategies and decreases public compliance with preparedness measures. Such exclusion not only undermines the effectiveness of interventions but also deepens existing social inequalities by perpetuating uneven access to resources and support. Conversely, platforms that support dialogue and community networks can help bridge the disconnect between communities and institutions, promoting mutual trust and more context-sensitive solutions.

Strong community engagement brings fresh insights and practical innovations while also increasing the legitimacy of DRM and CCA policies. With appropriate resources and support, local involvement can sustain interventions, reduce economic and social impacts and enhance long-term resilience to hazards (Mechler, Mochizuki & Hochrainer-Stigler 2016). Actively involving communities as key stakeholders thus strengthens advocacy, promotes knowledge sharing and ensures policy alignment with the needs of vulnerable populations.

### Implications of inadequate and donor-dependent funding

Inadequate funding remains a major barrier to the effective implementation of DRM and CCA in Sierra Leone. Financial limitations restrict the scope of interventions, often leaving the most vulnerable communities unsupported (World Bank 2016). Moreover, heavy reliance on short-term donor funding pressures implementing agencies to prioritise quick, visible outcomes over long-term, sustainable solutions contributing to cycles of temporary and often superficial interventions.

Effective DRM and CCA require substantial and sustained investment to strengthen preparedness, EWS and climate-resilient infrastructure. However, government funding remains insufficient and is often delayed, undermining the capacity of key institutions such as the NDMA and the NCCS. The absence of systematic, reliable data further hampers government budgeting and policy prioritisation, resulting in reactive rather than proactive measures.

While donor contributions provide critical technical and financial support, over-reliance reduces national ownership and may lead to fragmented, unsustainable efforts. In addition, bureaucratic red tape, corruption and stringent donor conditions delay the disbursement of funds and complicate timely responses ultimately weakening community resilience (ODI 2015).

To ensure long-term effectiveness, the government must allocate consistent domestic funding for DRM and CCA while also leveraging private sector partnerships. Such collaboration can enhance investment, encourage innovation and distribute risk more broadly, supporting a more sustainable and resilient national approach.

### Disaster risk management and climate change adaptation data availability, data sharing and disaster risk communication

Effective DRM and CCA depend heavily on the availability of reliable data, efficient data sharing and robust communication systems. These elements are essential for evidence-based decision-making, the dissemination of early warning and timely evacuation of vulnerable populations. Collectively, they enhance preparedness, coordination among agencies and the efficient allocation of resources, thereby supporting a unified disaster response.

In Sierra Leone, both the NDMA and the NCCS, though operating on a limited scale, have leveraged local media, social media platforms and CBOs such as FEDURP and CDMC. These efforts aim to raise awareness, counter misinformation and strengthen community resilience in line with the goals of the SFDRR and the Paris Agreement (UNFCCC 2015).

Localised communication remains crucial. Indigenous channels such as radio broadcasts, television segments and door-to-door campaigns ensure that at-risk communities receive timely and accurate information on disaster preparedness (Miles, Bang & Martin 2021). Furthermore, NDMA and NCCS have deployed tools such as the Climate Information Disaster Risk Management and Early Warning System (CIDMEWS), the Flood Anticipation Tool (FAT) and local language disaster dictionaries. These initiatives support community engagement, understanding and a sense of ownership over DRM and CCA efforts.

Programmes such as School Disaster Risk Education (SDRE), along with localised communication strategies, further bolster community preparedness. However, their effectiveness hinges on sustained funding, strengthened technical capacity and robust data-sharing frameworks.

Currently, limited access to data and persistent communication gaps hinders accurate risk assessments, timely responses and long-term resilience building, thereby exacerbating vulnerability and economic losses. Therefore, significant investment in data infrastructure and communication networks is imperative. Such investment would enable more coordinated responses, enhance technical capacities and improve the overall effectiveness of DRM and CCA strategies in Freetown and beyond (Sovacool & Linner 2017).

### Implications of the absence of a judiciary to adjudicate disaster risk management and climate change adaptation issues

The successful implementation of DRM and CCA in Sierra Leone hinges on the presence of strong legislative and judicial frameworks. However, there is currently no dedicated judiciary mechanism to address environmental and DRM- and CCA-related matters. The absence of such a system undermines the enforcement of policies, weakens institutional accountability and leaves room for corruption and inefficiency in the use of DRM and CCA resources.

Establishing a specialised environmental and DRM judiciary could play a pivotal role in enforcing compliance, addressing policy gaps and ensuring transparency. Such a body would also support the regular revision of legal instruments to reflect emerging risks and evolving climate realities, thereby strengthening the adaptive capacity and resilience of vulnerable communities (Wisner et al. 2004).

Furthermore, an effective judiciary is crucial for upholding international DRM and CCA commitments safeguarding human rights and amplifying the voices of marginalised populations (Knox 2016). In the current context, the lack of judicial oversight significantly impedes the capacity of institutions such as the NDMA and NCCS to fulfil their national mandates and international obligations.

Without legal mechanisms to address non-compliance and violations, vulnerable communities are exposed to recurring injustices and systemic exclusion. This erodes public trust in DRM and CCA interventions, diminishing civic engagement, compliance and the overall effectiveness of national resilience-building efforts.

### Implications of inadequate risk assessment, monitoring and evaluation mechanism on disaster risk management and climate change adaptation implementation

The findings reveal that both the NDMA and NCCS in Freetown, Sierra Leone, lack robust and comprehensive mechanisms for risk assessment, monitoring and evaluation. This deficiency significantly affects the effective implementation of DRM and CCA initiatives. Accurate and context-specific risk assessments are essential for identifying potential hazards and their impacts on vulnerable populations and critical infrastructure. These assessments play a vital role in informing EWS, guiding the allocation of resources and shaping evidence-based policy decisions (Birkmann et al. 2016). When risk assessments are inadequate or absent, disaster risks may be misjudged, resulting in poorly targeted interventions that fail to address the actual needs of at-risk communities and lead to inefficient or misdirected use of limited resources. Moreover, the absence of effective monitoring and evaluation mechanisms prevents systematic learning from past DRM and CCA interventions. As a result, strategies remain reactive and static, lacking the necessary adaptability to respond to evolving risks and changing environmental conditions. To ensure successful integration and implementation of DRM and CCA, it is imperative to strengthen these foundational systems for informed decision-making, continuous improvement and enhanced resilience outcomes.

### Implications of corruption

Corruption poses a major obstacle to the effective integration and implementation of DRM and CCA in Sierra Leone. Community reports indicate that corrupt practices weaken state institutions and hinder the enforcement of policies, with little accountability from policymakers. This endemic issue often results in the diversion of funds intended for DRM and CCA efforts, leaving communities under-resourced and compromising essential services such as EWS (Fenner & Mahlstein 2019).

Corruption not only erodes institutional capacity but also demotivates public sector staff, further weakening the execution of policies aimed at reducing disaster risks (Alexander 2017). As a result, community vulnerability increases, and public trust in state institutions declines. When citizens perceive governance structures as corrupt, their willingness to participate in DRM and CCA initiatives diminishes, leading to reduced policy compliance and civic engagement. Furthermore, corruption damages the relationship between state institutions and international donors. Misappropriation of funds and lack of transparency can lead to donors’ delay disbursements or imposing restrictive funding conditions, which undermine the timeliness and effectiveness of resilience-building programmes. In some cases, corruption may even extend to the manipulation of risk assessment data, compromising the reliability of information essential for planning, implementation and monitoring.

To address these challenges, the government of Sierra Leone must prioritise anti-corruption measures across all levels of governance. Strengthening accountability mechanisms, ensuring transparency in fund allocation and cultivating a culture of integrity are essential. Equally important is the empowerment of civil society and local communities to serve as watchdogs and whistleblowers, ensuring a participatory approach to governance that supports sustainable DRM and CCA outcomes.

### Implications of political influence and political instability

Achieving effective DRM and CCA in Sierra Leone requires strong and sustained political commitment. This includes the establishment of robust institutions, prioritisation of resource allocation and the enactment of supportive legislation aimed at enhancing community resilience (Islam et al. 2021). However, political influence can sometimes hinder rather than help. When short-term political interests are prioritised over long-term sustainability, it often leads to corruption, mismanagement of funds and weakened policy implementation (Islam et al. 2021).

Moreover, political rivalries and conflicting interests can disrupt coordination and strategic decision-making. Shifts in leadership often bring changes in policy priorities, undermining the consistency and continuity that are critical for successful DRM and CCA efforts (Nightingale 2017). In this context, political stability and unwavering commitment from Sierra Leone’s central government are vital. Without them, the integration of DRM and CCA into planning processes remains vulnerable to disruption, ultimately intervention efforts to build community resilience.

### Implications of community attitudes and behaviour

Achieving effective DRM and CCA in Freetown, Sierra Leone, requires significant shifts in both community attitudes and behaviours. The study highlights a persistent ‘blame game’ between state actors and local communities. Government institutions often attribute poor compliance to community reluctance, while communities, in turn, blame state agencies for neglect and exclusion from decision-making processes. This mutual mistrust affects effective implementation. Community engagement is critical for enhancing risk awareness, preparedness and resilience. However, when communities are excluded from DRM and CCA processes, they are more likely to resist state-led initiatives (Mendis et al. 2023).

Further complicating implementation is the lack of adequate support from the state. Many communities feel dis-empowered, particularly in informal settlements where land tenure insecurity leads to fears of eviction. This insecurity undermines local adaptation efforts, affecting the quality of housing and the stability of livelihoods (Mendis et al. 2023).

### Implications of inadequate research and technology

Effective DRM and CCA pivot on the availability and application of robust research and appropriate technologies. Research plays a critical role in providing the evidence base necessary for institutions such as the NDMA and NCCS to make informed policy decisions. It enables a better understanding of the socio-economic impacts of various interventions and supports the accurate identification of disaster risks.

In the absence of adequate research and reliable hazard data, policymaking becomes speculative and reactive, resulting in poorly designed, non-evidence-based strategies that often fail to address the real root causes of vulnerabilities of communities. Furthermore, limited access to technological resources undermines the effectiveness of EWS. Delays in issuing timely alerts to ‘at-risk’ populations not only compromise response times but also lead to increased casualties and obstruct efforts to build local resilience (Wisner et al. 2004).

### Implications of poor urban planning

The SFDRR sets a global target to significantly reduce disaster-related mortality by 2030. However, achieving this goal remains elusive in Freetown, Sierra Leone, where the city continues to face frequent and severe hazards such as flooding, landslides, fires and coastal erosion (UNDRR 2015). Rapid urbanisation has contributed to the unregulated expansion of informal settlements in high-risk areas, highlighting the critical need for more effective urban planning (Allen et al. 2017).

Although governmental responses and policy reforms have followed past disasters, these efforts often focus on short-term fixes rather than addressing underlying drivers of vulnerability. As a result, recovery remains slow, particularly for marginalised populations. Continued deforestation and unplanned land use while offering short-term economic gains have inadvertently heightened disaster risk, increasing the exposure and susceptibility of already vulnerable communities (Cui et al. 2019; Fullah 2025; Turay & Gbetuwa 2022).

### Key requirements for effective disaster risk management and climate change adaptation

To integrate and implement DRM and CCA successfully, a multi-dimensional and systemic approach is essential. This includes the following:

- Strengthening policy, legislative and institutional frameworks to ensure coordinated and sustained action.
- Demonstrating strong political will and government commitment to long-term resilience goals.
- Conducting robust risk assessments and enhancing EWS and monitoring capabilities.
- Promoting integrated planning and policy development, fostering collaboration across sectors and stakeholders.
- Engaging communities meaningfully in risk reduction processes and building their capacities.
- Establishing adequate financial mechanisms, including post-disaster insurance schemes and targeted incentives.
- Investing in resilient infrastructure, such as durable housing, functional drainage systems, reliable road networks, efficient solid waste management systems and effective flood defences.
- Supporting community-led initiatives, such as afforestation and mangrove restoration, to reduce environmental risks and promote ecological resilience.

By addressing both structural and social vulnerabilities, these strategies can help reduce disaster risks, enhance adaptive capacity and promote sustainable recovery from climate-related disasters in Freetown.

## Conclusion

This study assessed the effectiveness of DRM and CCA policies in Freetown, Sierra Leone. The findings highlight that implementation success is shaped by a complex interplay of interconnected factors, with socio-economic vulnerabilities emerging as critical underlying drivers. Effective DRM and CCA require sustained political will, supported by strong leadership from state institutions such as NDMA and NCCS, through the EPA-SL. To enhance resilience at the local level, it is essential to prioritise resource allocation, strengthen institutional capacity and strengthen meaningful community engagement. These actions will enable the government to align national efforts with global frameworks such as the Sendai Framework for Disaster Reduction and the Paris Agreement, contributing to more resilient and adaptive communities by 2030.

The following action proposals outline essential strategies for enhancing the effectiveness of DRM and CCA in Sierra Leone.

Firstly, there is an urgent need to establish and enforce a comprehensive national framework that integrates DRM and CCA. This should be accompanied by harmonised policies that prioritise community engagement and utilise local languages to ensure accessibility and inclusiveness.

Secondly, to support these efforts, the development of policy support facilities (PSF) is recommended. These facilities would help coordinate collaborative, proactive DRM and CCA initiatives. Furthermore, the creation of detailed vulnerability profiles and hazard maps will enable targeted and evidence-based interventions.

Thirdly, addressing legal issues to DRM and CCA also requires attention. The establishment of a specialised judiciary or environmental court system is crucial for resolving disputes efficiently and with technical expertise.

Fourthly, capacity building and institutional strengthening must be prioritised to ensure that DRM and CCA strategies are effectively implemented. The government should develop a sustainable national financing mechanism such as pre-disaster contingency funds and post-disaster insurance schemes while enforcing strict anti-corruption measures to guarantee transparent and accountable resource management.

Fifthly, the creation of a centralised, open-access data platform is critical for promoting data-driven decision-making in DRM and CCA planning and response, while the effective implementation of DRM and CCA policies in Sierra Leone depends critically on the inclusive participation of local communities. To strengthen public awareness and responsiveness, the NDMA and NCCS should develop a comprehensive national disaster risk communication strategy. This should include the establishment of an Integrated National Public Alert and Warning System (INPAWS) and the reinforcement of a robust monitoring and evaluation mechanism to assess the effectiveness of implemented strategies.

Sixthly, to ensure continuity and impartiality in DRM and CCA initiatives, it is essential that the government safeguards the political neutrality of relevant institutions through appropriate legislation and policy frameworks.

Seventhly, the NDMA and NCCS, via the EPA-SL, must prioritise education and outreach programmes to encourage a culture of proactive risk management. This should be complemented by the promotion of sustainable land use and urban planning practices to reduce vulnerability to future hazards. Above all, sustained and meaningful community engagement must remain a cornerstone of DRM and CCA efforts in Sierra Leone.

## Footnotes

1 Sam-Mbomah, E. 2024: https://doi.org/10.26174/thesis.lboro.28211306.
